# Syntheses, Characterization, and Antioxidant Evaluation of Cu^2+^, Mn^2+^, and Fe^3+^ Complexes with a 14 Membered EDTA-Derived Macrocycle

**DOI:** 10.3390/molecules24193556

**Published:** 2019-10-01

**Authors:** Yedith Soberanes, Rosa Elena Navarro, Motomichi Inoue, Enrique F. Velázquez-Contreras, Melissa Beltran Torres, Gustavo Lugo, Rogerio R. Sotelo-Mundo, Alex J. Salazar-Medina

**Affiliations:** 1 Departamento de Investigación en Polímeros y Materiales, Universidad de Sonora, Hermosillo Sonora 83000, Mexico; yedith16@gmail.com (Y.S.); rnavarro@guaymas.uson.mx (R.E.N.); motomichiinoue@cox.net (M.I.); evlzqz@guaymas.uson.mx (E.F.V.-C.); melissabt21@gmail.com (M.B.T.); 2 Laboratorio de Estructura Biomolecular, Centro de Investigación en Alimentación y Desarrollo A.C., Hermosillo, Sonora 83304, Mexico; rrs@ciad.mx; 3 Departamento de Ingeniería Química, Universidad de Sonora, Hermosillo, Sonora 83000 Mexico; gustavolugop@gmail.com; 4 Cátedras CONACYT-Departamento de Investigación en Polímeros y Materiales, Universidad de Sonora, Hermosillo, Sonora 83000, Mexico

**Keywords:** transition metal complexes, macrocycle, coordination chemistry, theoretical calculation, antioxidant

## Abstract

The Cu^2+^, Mn^2+^, and Fe^3+^ complexes of a 14 membered macrocycle were synthesized and their antioxidant capacities were evaluated against ABTS and DPPH radicals, with the objective of collecting insights into the biomimetic role of the central metal ions. The macrocycle, abbreviated as H_2_L14, is a derivative of EDTA cyclized with 1,4-diamine, and the moderately flexible macrocyclic frame permits the formation of [ML14·H_2_O] chelates with octahedral coordination geometries common among the metal ions. The metal complexes were characterized by electrospray-ionization mass spectrometry, Fourier transform infrared spectroscopy, and Raman and X-ray photoelectron spectroscopic methods, as well as thermogravimetric analysis; the octahedral coordination geometries with water coordination were optimized by DFT calculations. The antioxidant assays showed that [FeL14·H_2_O]^+^ was able to scavenge synthetic radicals with moderate capacity, whereas the other metal chelates did not show significant activity. The Raman spectrum of DPPH in solution suggests that interaction was operative between the Fe^3+^ chelate and the radical so as to cause scavenging capability. The nature of the central metal ions is a controlling factor for antioxidant capacity, as every metal chelate carries the same coordination geometry.

## 1. Introduction

The prevention or reduction of cell damage caused by free radicals is of great current and rising concern. With the intention of solving this problem, a wide variety of synthetic antioxidants and radical scavengers have been developed in pharmaceutical and nutritional fields [1]. Homeostasis against the action of free radicals and active oxygen species is maintained in cells by a specialized antioxidant network, where superoxide dismutase converts O_2_^●−^ to H_2_O_2_ or molecular oxygen. The successive conversion of H_2_O_2_ to water and oxygen is catalyzed by catalase and peroxidase enzymes [2,3]. A common feature among these metalloenzymes is the presence of transition metal ions such as Cu^2+^, Mn^2+^, Ni^2+^, and Fe^3+^ ion as active centers [4].

**Figure 1:**
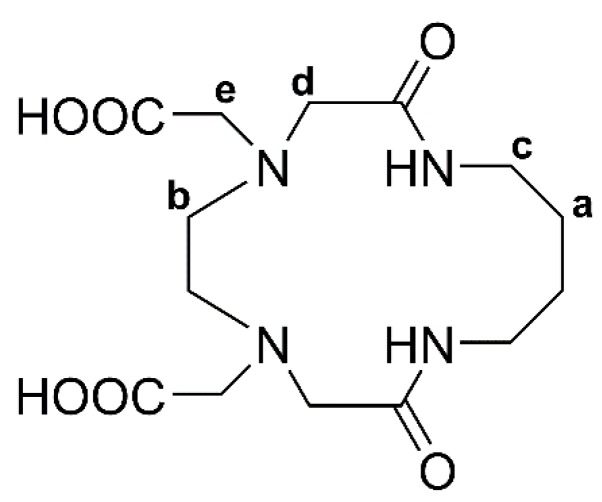
Scheme of the 14 membered macrocycle, 2,9-dioxo-1,4,7,10-tetraaza-4,7-cyclotetradecanediacetic acid, abbreviated as H_2_L14, with acidic protons. The CH_2_ protons are labeled for NMR assignments.

In our previous works, the antioxidant activity and mimetic were evaluated for a series of binuclear Cu^2+^ and Fe^3+^ complexes with cyclophanes derived from EDTA and aromatic diamines, and all the complexes were found to be capable of inhibiting ABTS (2,2′-azino-bis(3-ethylbenzothiazoline-6-sulfonate) disodium salt) free radicals with high activity comparable to that of ascorbic acid [5,6]. The chelating EDTA moiety in the cyclophanes permits the metal complexation with sufficient stability to avoid the pro-oxidant effects caused by metal ions in their free state, and the activity is expected to be enhanced by the binuclear metal center surrounded by three types of donor sites: carboxylate, amino, and amide groups. In line with these results and with the objective of studying the effects of the metal centers, the present work examined the antioxidant capacity of the mononuclear Cu^2+^, Fe^3+^, and Mn^2+^ chelates that had identical coordination geometries. The selected ligand was a 14 membered aliphatic macrocycle (abbreviated as H_2_L14) shown in Figure 1. The framework of this macrocycle is moderately flexible so that the Ni^2+^ complex forms a [NiL14·H_2_O] molecule with well-defined octahedral coordination in the X-ray structure (Figure S1 in the Supplementary Materials); the same type of geometry is maintained for Cu^2+^ as well as Ni^2+^ in solution [7]. The antioxidant activities of the metal complexes are therefore expected to be controlled by the nature of the central metal ions. This paper reports the characterization of the three metal complexes in relation to their antioxidant activities.

## 2. Results and Discussion

### 2.1. X-ray Crystal Analysis of H_2_L14

**Figure 2:**
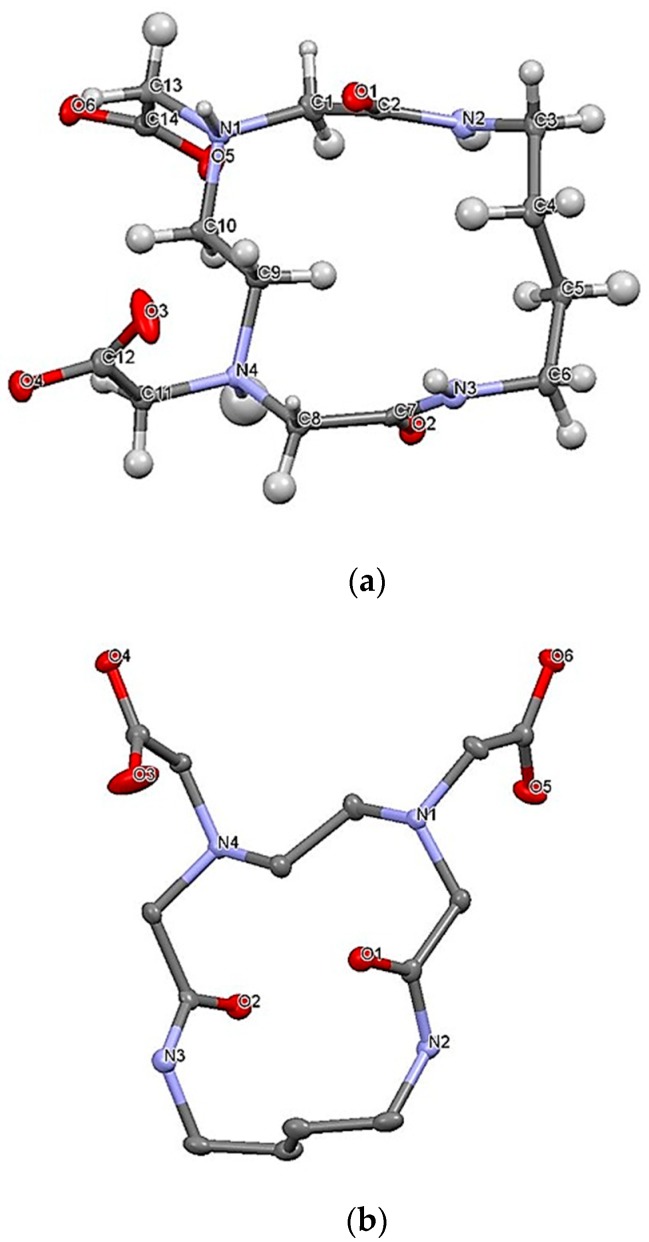
(**a**) Perspective view of the molecular structure of H_2_L14—atoms are drawn at the 50% probability level; (**b**) view from a different direction without hydrogen atoms for clarity.

The formation of macrocycle H_2_L14 shown in Figure 1 was confirmed by ^1^H NMR and mass spectroscopy. The conclusive confirmation was obtained by X-ray crystal analysis. The structure is presented in Figure 2, and the crystal data collection and structure determination are summarized in Tables S1–S6.

The ligand molecule was described by quasi-C_2_ symmetry about the axis through the C4–C5 and C9–C10 bonds (Figure 2). In the carboxylate groups, the C12–O4 and C14–O6 bonds were longer than the C12–O3 and C14–O5 bonds. Each molecule had a zwitterion structure, in which protonated atoms form intermolecular hydrogen bonds with the non-protonated atoms of the neighboring molecule with N4-H4∙∙∙O6′ and N1′-H1′∙∙∙O4 to construct a trimeric aggregate, as seen in Figure S2 and Table S7. Protonated and deprotonated N-acetate groups were present in the H_2_L14 receptor, supporting the proton population of 0.5 at each donor atom. The formation of a similar zwitterion structure was confirmed by ^1^H NMR in solution (Figure S3). Around pD 9, protons b, d, and e adjacent to the amino nitrogen underwent a large shift to higher δ values with decreasing pD, indicating that the first protonation equilibrium occurred at the amino nitrogen. With a further decrease of pD below ~5, proton d showed a second shift due to the protonation at the carboxylate oxygen.

### 2.2. Characterization of Cu^2+^, Mn^2+^, and Fe^3+^ Complexes

Reactions between Cu^2+^, Mn^2+^, and Fe^3+^ salts and H_2_L14 receptor gave the complexes [CuL14]^0^, [MnL14]^0^, and [FeL14]^+^, respectively, designated henceforth as CuL14, MnL14, and FeL14. The complexes were characterized by ESI-MS, thermogravimetric analysis (TGA), and vibrational spectroscopy. The thermal behaviors of CuL14, MnL14, and FeL14 were studied by TGA to determine the presence of coordinated water molecules. The thermograms are shown in Figures S5–S7 in the Supplementary Materials, and the decomposition steps and assignments are described in Table 1.

The thermogram of CuL14 (Figure S5) shows the stepwise release of two water molecules at 71 and 114 °C, and the weight loss at 168 °C is ascribable to a water molecule coordinated to the central metal. The release of a couple of acetate pendant groups from the EDTA moiety finished at 255 °C. The weight-loss pattern described by MnL14 indicates the presence of a water molecule included in the coordination sphere and liberated at 197 °C (Figure S6). The Fe^3+^ complex, FeL14, involved three water molecules: two of them involved in the crystal lattice were released at 117 °C, and the third in the coordination sphere at 182 °C (Figure S7). Additional weight loss at 245 °C was due to counter ion NO_3_, and carboxylic pendant groups were eliminated at 405 °C.

After the release of water molecules, all the complexes started the decomposition of the receptor in a similar fashion. The residual metal oxide formed after thermal treatment was employed to calculate metal content in each complex. For all metal complexes, the experimental values were in good agreement with the calculated values based on the number of water molecules detected.

**Figure 3:**
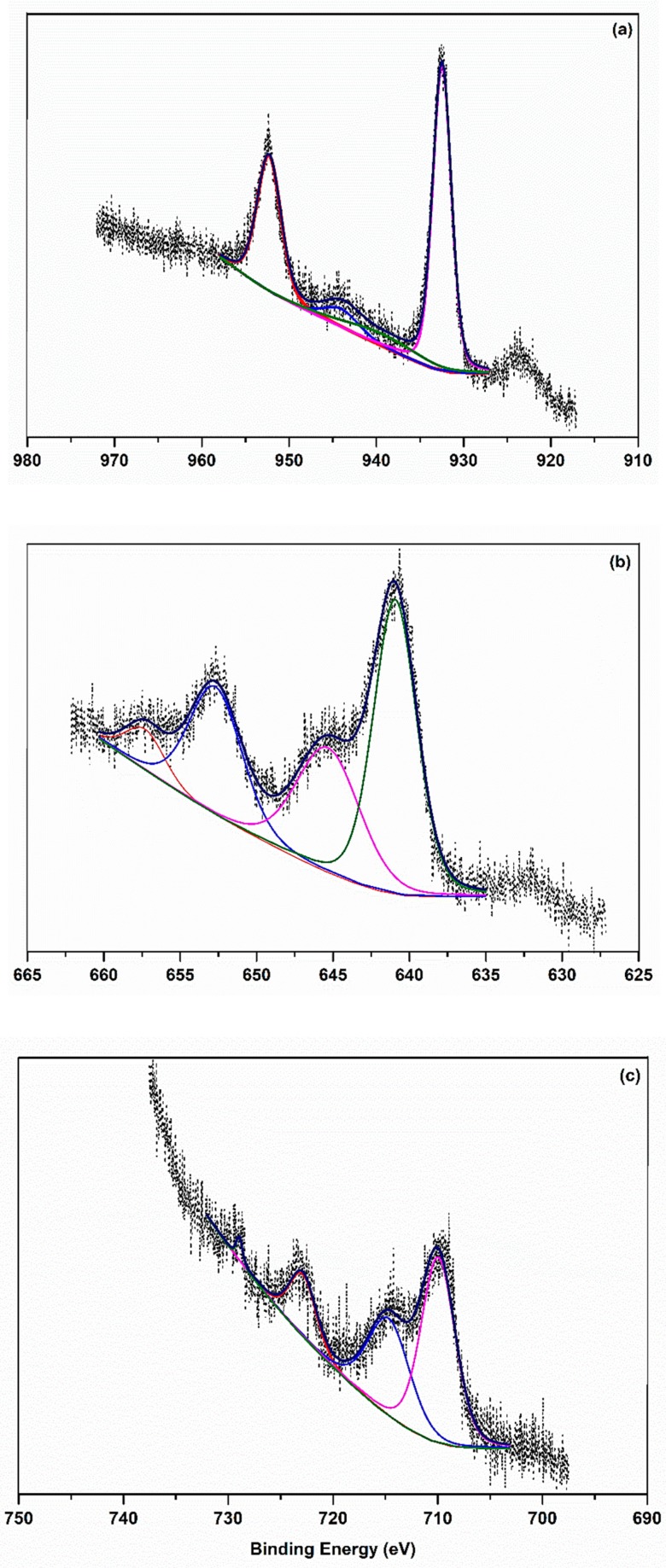
XPS spectra and fitted curves of (**a**) CuL14, (**b**) MnL14, and (**c**) FeL14 metal complexes.

The XPS spectrum of MnL14 showed 2p_3/2_ and 2p_1/2_ core electron peaks at 640.9 and 652.6 eV, respectively (Figure 3). These values agreed with those reported previously for bivalent Mn [8,9]. In the case of FeL14, the peaks corresponding to 2p_3/2_ and 2p_1/2_ were found at 710.0 and 722.9 eV (Figure 3), which are attributed to trivalent iron [8,10,11,12]. For the copper spectrum of CuL14, the well-defined peaks at 932.5 and 952.3 eV are attributed to the 2p_3/2_ and 2p_1/2_ core electron levels (Figure 3). Every peak was accompanied by a broad peak at the higher energy side. The latter is attributable to the shake-up satellite that appears characteristically in transition metals with an open 3d-eleclecton shell [12,13,14]. In some cases, the satellites were almost as intense as the main peaks, as observed for the 2p_3/2_ peaks of the Mn and Fe (d^5^) complexes. These shake-up satellites that appeared in the core electron spectra of the transitional metal complexes resulted from a charge–transfer-type transition. Charge-transfer shake-up satellites are reasonably expected for the core-electron spectra of transition metals in complexes having a π bond character, which induces a ligand-to-metal charge transfer. The positions and intensities of these type of satellites are defined by the nature of the metal, ligands, and the symmetry of the complex [14]. Binding energies of the core electron peaks of the L14 metal complexes are summarized in Table 2.

The infrared and Raman spectra of the H_2_L14 receptor and the CuL14, MnL14, and FeL14 complexes exhibited similar spectral patterns (Figures S9–S12). Table 3 shows the assignments of the main bands related to coordination sites of the receptor (N–H ν, C=O ν, C=O ν of amide I and C=O δ from amide II) as well to coordinated water (H–O–H ν).

The intense, sharp N–H ν band of H_2_L14 between 3320 and 3350 cm^−1^ reduced its intensity after Cu^2+^ coordination and became broad by the formation of the Mn^2+^ and Fe^3+^ complexes even to overlap the C–H ν band at 2930–2945 cm^−1^. The broadening of these bands and the appearance of an additional broad band around 3300–3400 cm^−1^ or an even smaller wavenumber are attributable to the presence of water molecules and the consequent hydrogen bonding. The sharp C=O ν band of amide I (at 1644 cm^−1^) of the H_2_L14 receptor in Raman spectra was slightly weakened in the Cu^2+^ complex, and additionally broadened in the Mn^2+^ and Fe^3+^ complexes, as a consequence of the participation of amides in the coordination of the metals to the receptor.

The presence of water molecules (in coordinated and uncoordinated states) in the metal complexes induced the broadening of the bands at ~3400 cm^−1^. The axial coordination of a water molecule to each metal center was supported by the observation of bands at about 500 cm^−1^.

### 2.3. Theoretical Calculations

**Figure 4:**
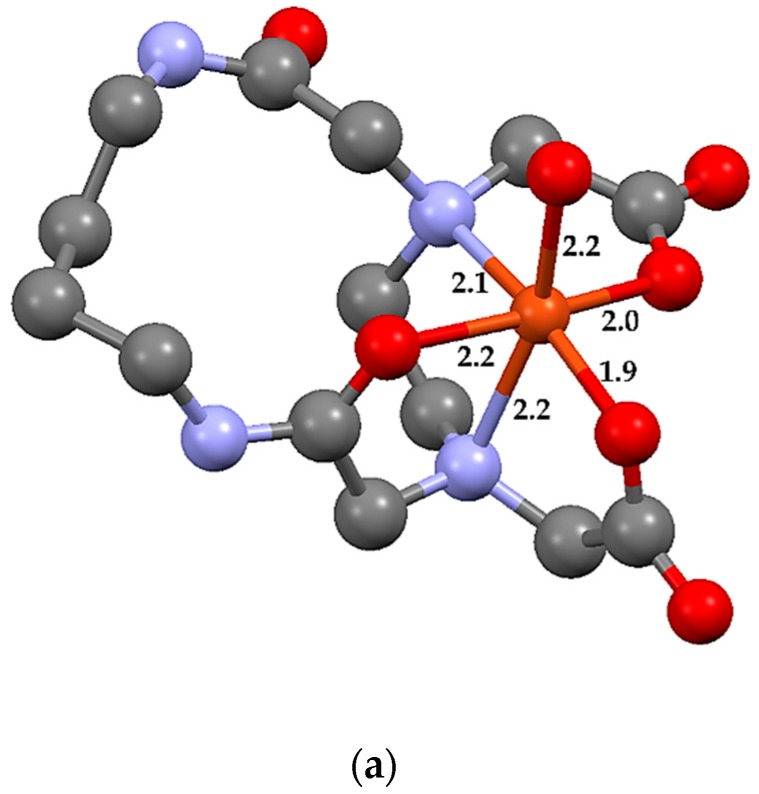

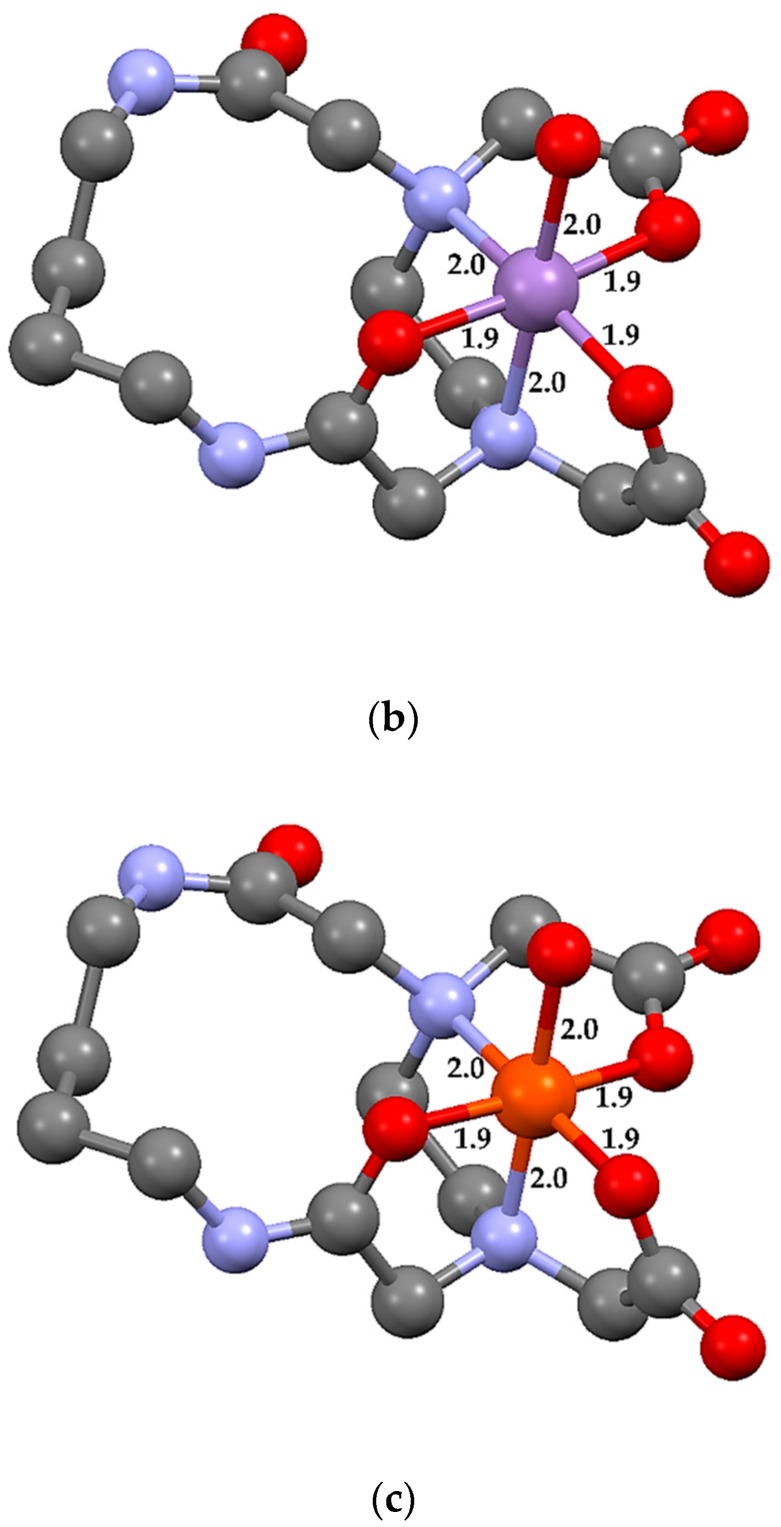
Optimized structures of (**a**) CuL14·H_2_O, (**b**) MnL14·H_2_O, and (**c**) FeL14·H_2_O molecules by DFT calculation. The bond lengths around the metal centers are presented in Å.

The DFT method was used to predict the ground state structures of metal complex molecules [ML14·H_2_O] (M = Cu^2+^, Mn^2+^, and Fe^3+^) at the LANL2MB/6-31G (d, p) level. The X-ray structure of [NiL14·H_2_O] was employed as the starting point in the calculation. Table 4 contains selected geometric parameters for the optimized structures presented in Figure 4. It emerged that the calculated bond lengths and angles were in a very good agreement with those of the X-ray crystal structure of [NiL14·H_2_O], which is presented in Figure S1 for comparison [7]. Frequency calculations on the equilibrium geometries showed no imaginary frequencies for all the structures with the only exception of the MnL14 complex, for which complete suppression of an imaginary frequency due to the presence of hydrogen vibrations was not possible.

### 2.4. Chemical Antioxidant Assays

The antioxidant activity was evaluated as the half-maximal inhibitory concentration (IC_50_) against ABTS (2,2′-azino-bis(3-ethylbenzothiazoline-6-sulfonate) disodium salt) and DPPH (2,2-diphenyl-1-picrylhydrazyl) radicals. According to the results, as summarized in Table 5, the Fe complex shows the highest antioxidant activity with IC_50_ values of 153 ± 6 µM and 193 ± 5 µM against ABTS and DPPH radicals, respectively. The activities of the other compounds were too small to determine IC_50_ values. For this reason, the inhibition percentages at a concentration of 200 µM were compared in Table 5, which showed that the free ligand exhibited a higher inhibition capacity against ABTS than the Cu and Mn complexes, despite the lack of a metal center.

**Figure 5:**
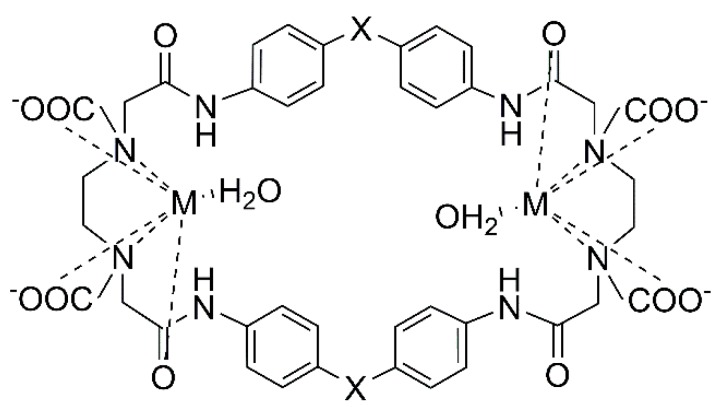
Structure of macrocyclic binuclear metal complexes: Cu_2_PO and Fe_2_PO (X = ether oxygen); Cu_2_PC and Fe_2_PC (X = methylene). M = Cu^2+^ or Fe^3+^.

High activities against ABTS were reported for macrocyclic binuclear metal complexes: IC_50_ = 10 ± 0.6 µM and 18 ± 0.5 µM for Cu^2+^ complexes Cu_2_PO and Cu_2_PC (Figure 5), respectively [6], and 38 ± 0.9 µM and 28 ± 0.2 µM for Fe^3+^ complexes Fe_2_PO and Fe_2_PC (Figure 5) [15]. These complexes are inhibitors as powerful as ascorbic acid (IC_50_ = 14.9 ± 0.5 µM), although the inhibitory capacities are moderate when compared with glutathione (<12.5 µM) and caffeic acid phenethyl ester (<12.5 µM). The binuclear core may be one of the factors for the efficiency, as suggested by the lower activities of the L14 complexes.

The DPPH assay is not suitable for binuclear Cu^2+^ complexes due to the formation of a colored complex, and, for the binuclear Fe^3+^ complexes, the reduced activity against this radical was not enough to calculate IC_50_. By contrast, IC_50_ of FeL14 against DPPD was nearly identical with the value against ABTS. The mononuclear complex of the reduced size was expected to reach the radical site of DPPH closely enough as to enhance the activity in comparison to the binuclear ones. This possible effect was found only for the FeL14 among the tested L14 complexes. Since the DFT-generated structures involved almost the same bond lengths and angles independently of the nature of the central metals, the significant activity shown only by the Fe complex is ascribable to the electronic density of the metal center rather than a conformational arrangement of chelate molecules.

**Figure 6:**
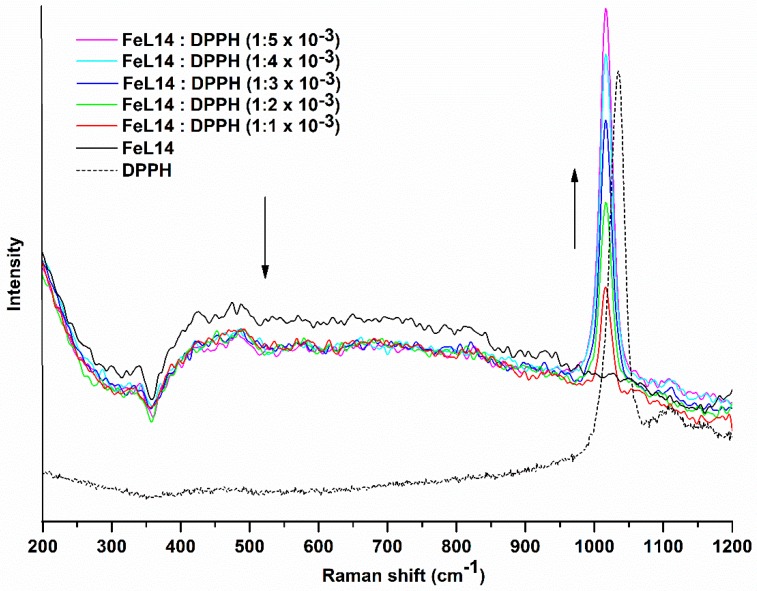
Decrease in the intensity of the M–OH_2_ stretching band (550 to 590 cm^−1^) of the FeL14 complex by addition of the DPPH free radical. Shift of the aromatic C–H in-plane bending band of the DPPH radical in the presence of the FeL14 complex and signal enhancement proportional to the DPPH concentration.

Possible interactions between the metal complexes and DPPH radical were studied by Raman spectroscopy in solution. The aromatic C–H in-plane bending band of DPPH radicals observed at 1035 cm^−1^ was shifted to 1018 cm^−1^ in the presence of FeL14 complex (Figure 6). The same type of shift was observed for the Cu and Mn complexes as well as for the free ligand L14 (Figures S12–S14). As for the effect of DPPH radical on the Fe complex, the M–OH_2_ stretching band, which appears between 550 and 590 cm^−1^ in solution as well as in solid state, was weakened with the augmentation of the mole ratio of DPPH (Figure 6), unlike CuL14 and MnL14 complexes (Figures S13 and S14. This phenomenon may be related to the fact that only the Fe^3+^ complex was capable of deactivating DPPH free radicals.

## 3. Materials and Methods

### 3.1. Synthesis and X-ray Crystal Analysis of H_2_L14

The macrocycle H_2_L14 was synthesized by a cyclization reaction between 1,4-diaminobutane and EDTA dianhydride, O(OCCH_2_)_2_N(CH_2_)_2_N(CH_2_CO)_2_O, according to the methodological procedure reported previously in Reference [7]. The formation and purity were confirmed by the melting/decomposition point, electrospray-ionization (ESI) mass spectrometry, ^1^H-NMR, and single-crystal X-ray analysis. Single crystals of L14 were grown by slow evaporation from an ethanol solution of the receptor in a glass vial. The X-ray diffraction data were collected on a Bruker D8 QUEST diffractometer system, equipped with a multilayer mirror monochromator and a Cu Kα microfocus sealed tube (λ = 1.54178 Å) at 120.0 K. The absorption effects were corrected by the multi-scan method (SADABS). Direct methods [16] were employed to solve the structure through the ShelXT [17] structure solution program, and the refinement was performed with the ShelXL [18] refinement package with least squares minimization. Both programs were implemented in the Olex2 software. The crystal was modelled as a non-merohedral twin with the volume ratio of two twin domains refined at 67.8:32.2, and the structure analysis was performed with Mercury 3.9 [19]. Crystallographic data for the H_2_L14 receptor were deposited at the Cambridge Crystallographic Data Centre as Supplementary Publication no. 1950325.

### 3.2. Syntheses of Cu^2+^, Mn^2+^, and Fe^3+^ Complexes

All employed reagents and solvents were of analytical grade. The complexes were obtained by mixing aqueous solutions of H_2_L14 receptor with appropriate meal salts, CuCO_3_·Cu(OH)_2_, MnCO_3_, and Fe(NO_3_)_3_·9H_2_O in a molar ratio 1:1. The carbonate insoluble fraction was removed by filtration. The solution was concentrated and acetone was added to precipitate the metal complexes, which were separated by filtration and dried at 50 °C under vacuum. CuL14·3H_2_O: Yield: 90 %. M. p.: 230 °C (decomposed). MS (ESI^+^) *m*/*z*: 406.2 (100 %) [(C_14_H_22_N_4_O_6_Cu) + H]^+^. Anal. Cu, 14.4%; calc 13.8%. MnL14·H_2_O: Yield: 88%. M. p.: 375 °C (decomposed). MS (ESI^+^) *m*/*z*: 398.1 (100%) [(C_14_H_22_N_4_O_6_Mn) + H]^+^. Anal. Mn, 13.4 %; calc 13.2%. FeL14·NO_3_·3H_2_O: Yield: 85%. M. p.: 300 °C (decomposed). MS (ESI^+^) *m*/*z*: 398.1 (16%) [C_14_H_22_N_4_O_6_Fe]^+^. Anal. Fe, 12.1%; calc 10.9%.

### 3.3. Physical Measurements

The thermogravimetric analyses (TGAs) of the metal complexes were carried out with a Perkin–Elmer Pyris 1 TGA apparatus at a heating rate of 5 °C/min for CuL14 and MnL14, and 2.5 °C/min for FeL14 in a temperature range of 25 to 800 °C under an O_2_ atmosphere at a flow rate of 20 mL/min. Spectrum evaluations were performed using the Pyris version 11.1.1.0492 software package.

The infrared spectra were recorded in the spectral range between 4000 and 250 cm^−1^ in KBr pellets with a Perkin–Elmer FTIR/FIRS Spectrometer Frontier instrument. A total of 32 scans were performed. Raman spectra were obtained using a HORIBA LabRam HR Evolution Raman microscope at room temperature. A He–Ne laser was employed with an energy of 633 nm from 20 mW to 100 mW and a slit of 100 µm. The X-ray photoelectron spectra (XPS) were obtained on a Perkin–Elmer PHI 5100 model with a Mg Kα source under a vacuum of 4 × 10^−9^ torr. All the spectra were corrected to alkyl type carbon C1s (285.0 eV) and fitted by applying a Tougard baseline and a Gaussian–Lorentzian function to each peak.

### 3.4. Geometry Optimization

The DFT calculations on [ML14·H_2_O] (Cu^2+^, Mn^2+^, and Fe^3+^) were carried out with the Gaussian 09 program [20]. The long-range corrected hybrid density functional with damped atom–atom dispersion corrections wB97XD [21] was used together with the LANL2MB relativistic corrected pseudopotentials and basis sets [22,23] for the metal atoms and the 6-31G (d,p) Gaussian basis set for the H_2_L14 macrocycle. The polarizable continuum model (PCM) [24,25] was used to include water as solvent.

### 3.5. Chemical Antioxidant Assays

The chemical antioxidant capacities of H_2_L14 and its Cu^2+^, Mn^2+^, and Fe^3+^ complexes were studied based on the original procedures of the ABTS [26] and DPPH [27] assays, with slight modifications. A dose–response curve was constructed as a function of the concentration of each complex, and the half-maximal inhibitory concentrations (IC_50_) were determined. The concentration of ABTS in ethanolic solution was adjusted so that the absorbance was 0.7 ± 0.02 at 754 nm, and the concentration of a DPPH methanolic solution was adjusted to show the absorbance 0.7 ± 0.02 at 515 nm. First, 245 µL of the ABTS radical solution was placed in every well of a 96 well microplate (Costar^®^, USA). Then 5 µL of sample solutions at concentrations 12.5, 25, 50, 100, and 200 µM were added to wells. After 5 min of incubation, the absorbance of each tested solution was read at 754 nm on an Omega spectrophotometer (BMG Labtech Inc., Ortenberf, Germany). For DPPH, 280 µL of the solution was placed in each well of the microplate, and 20 µL of sample solutions of 12.5, 25, 50, 100, and 200 µM were added to different wells. After 30 min of incubation, the absorbance was read at 515 nm.

## 4. Conclusions

The formation of the 14 membered macrocycle, 2,9-dioxo-1,4,7,10-tetraaza-4,7-cyclotetradecanediacetic acid, abbreviated as H_2_L14, was confirmed by the X-ray analysis, in addition to the previous studies of the Ni^2+^ complex. The Mn^2+^, Cu^2+^, and Fe^3+^ complexes were characterized by spectroscopies, and the chelate molecules [ML14·H_2_O] were confirmed, by DFT calculations, to have hexa-coordinated octahedral geometries with a close resemblance to the Ni^2+^ complex molecule in the bond lengths and angles. Among the metallic complexes and the uncoordinated receptor, the Fe^3+^ complex was capable of deactivating the synthetic free radicals ABTS and DPPH, with a moderate activity in reference to well-established antioxidants such as ascorbic acid and glutathione.

Comparison with the analogous binuclear metal chelates indicates that the effectiveness of a metal complex on deactivating free radicals is ascribable to a combined effect of the number and electronic density of the metal centers, rather than the coordination mode of the synthesized macrocycles.

## Figures and Tables

**Table 1:** Thermogravimetric analysis of H_2_L14 and its metal complexes.

	H_2_O		NO_3_	–CH_2_COO–	Metal %
Compound	Moisture ^1^	Coordinated ^1^	Counter Ion ^1^	Pendant Groups ^1^	Experimental/Theoretical
H_2_L14	n/a	n/a	n/a	19.5/286	n/a
				19.6/386	
CuL14·3H_2_O	4.2/71	3.9/168	n/a	23.6/255	14.4/13.8
	5.1/114				
MnL14·H_2_O	n/a	4.6/197	n/a	n/a	13.4/13.2
FeL14·NO_3_·3H_2_O	7.6/117	4.0/182	12.0/245	18.5/405	12.1/10.9

^1^ Data presented as percentage over temperature in Celsius degrees (%/°C).

**Table 2:** Binding energies (eV) of the core electron peaks of the L14 metal complexes.

Compound	2p_3/2_	2p_3/2_ (sat)	2p_1/2_	2p_1/2_ (sat)	Peak Differences 2p_3/2_−2p_1/2_
CuL14	932.5	939.5	952.3	944.0	19.8
MnL14	640.9	645.3	652.6	657.3	11.7
FeL14	710.0	714.7	722.9	728.9	12.9

**Table 3:** The IR and Raman bands observed for receptor H_2_L14 and its metal complexes in the 400–4000 cm^−1^ region.

Assignment	H_2_L14		CuL14·3H_2_O		MnL14·H_2_O		FeL14·3H_2_O	
	FTIR	Raman	FTIR	Raman	FTIR	Raman	FTIR	Raman
N–H ν	3348 vs	3351 m	3318 m	3317 vw	3229 s	3238 vw	3236 s	3238 vw
C–H ν	2933 s	2937 vs	2941 m	2931 vs	2935 s	2937 vs	2941 m	2943 m
C=O ν	1719 s	1723 w	-	-	1739 shd	1758 w	-	1772 w
O–C ν	1237 s 1285 s	1206 m	1316 m	1288 m 1322 m	1279 s 1328 s	1302 m	1292 shd 1243 shd	1295 m
C=O ν (amide I)	1661 s	1644 m	1655 s	1651 w	1673 s	1651 w	1642 s	1625 s
C=O δ (amide II)	1533 s	1534 w	1564 s	1584 m	1576 s	1550 vw	1576 shd	1537 shd
C–N ν	1150 m	1159 w	1312 m	1104 w	1113 m	1127 w	1097 w	1117 w
CO_2_–M ν	-	-	1655 s	1651 w	1619 vs	1651 w	1570 m	1618 m
M–N ν	-	-	493 w	468 m	451 w	462 m	463 vw	482 w
H–O–H ν cw	-	-	3414 m	3324 w	3476 shd	-	3457 shd	3331 w
M–OH_2_ ω	-	-	560 w	569 w	524 m	576 w	590 w	562 vw

M = Cu^2+^, Mn^2+^ or Fe^3+^. Abbreviations: ν, stretching; δ, bending; ω, wagging; vs, very strong; s, strong; m, medium; w, weak; vw, very weak; brd, broad; shp, sharp; shd, shoulder; cw, coordinated water.

**Table 4:** Bond lengths (Å) and angles (°) for [ML14·H_2_O] molecules.

Bond Type	CuL14·H_2_O	MnL14·H_2_O	FeL14·H_2_O	NiL14·H_2_O ^1^
D M–O(CO)	2.0 1.9	1.9 1.9	2.0 2.0	2.0 2.1
D M–O(CN)	2.1	1.9	2.1	2.1
D M–OW	2.2	2.0	2.1	2.0
D M–N	2.2 2.1	2.0 2.0	2.1 2.2	2.1 2.2
<O(CO)–M–O(CO)	92.7	90.5	91.7	92.5
<O(CO)–M–O(CN)	173.2 92.9	174.7 94.5	174.2 94.1	1709 95.1
<O(CO)–M–O_W_	101.5 98.5	90.1 94.0	84.3 96.3	93.1 98.1
<O(CO)–M–N	98.2 83.9 81.4 166.5	92.1 84.2 85.5 172.7	97.1 82.1 82.7 165.9	93.9 81.4 80.5 164.3
<N–M–O(CN)	78.7 89.8	86.6 90.6	82.6 92.1	82.5 90.0
<N–M–O_W_	160.3 95.0	177.7 91.2	178.2 95.7	172.9 96.8
<N–M–N	86.1	89.6	85.5	85.4

Oxygens from a carboxylic group are represented as –O(CO); oxygens from an amide group are represented as –O(CN), and oxygens from water are represented as OW. ^1^ Reference [7].

**Table 5:** Chemical antioxidant activity of H_2_L14 and its metal complexes. Inhibition percentages at the concentration of 200 µM, and half maximal inhibitory concentration (µM).

	ABTS		DPPH	
	Inhibitory %	IC_50_	Inhibitory %	IC_50_
H_2_L14	33 ± 0.8	n/a	2 ± 0.6	n/a
CuL14	21 ± 3.2	n/a	4 ± 15	n/a
MnL14	8 ± 1.6	n/a	9 ± 1.5	n/a
FeL14	63 ± 1.9	153 ± 6	52 ± 2.4	193 ± 5

Data are expressed as the mean of three independent experiments performed in triplicate. Ascorbic acid was used as a positive control (IC_50_/µM = 14.9 for ABTS, 17.7 for DPPH).

## Supplementary Materials

The following are available online, Figure S1: Crystal structure reported for NiL14 complex. Carbons are represented in gray, oxygens in red, nitrogens in violet, and nickel in green (Inoue, 1998) [7], Figure S2: Trimer formed by hydrogen bonds in the molecular structure of H_2_L14. Atoms are drawn at the 50% probability level, Figure S3: pD dependence of ^1^H NMR chemical shifts, δ referenced to DSS, of the H_2_L14 receptor. The solid lines are drawn as an aid to visualizing the trends in the chemical shifts. Minimal amounts of KOD and DCl were used to adjust pD, Figure S4: Thermal decomposition analysis of H_2_L14 receptor in solid state. The marks in solid lines show the weight loss percentage and the temperature at each decomposition step. The dashed line presents the derivative of weight loss (%/min), Figure S5: Thermal decomposition analysis of CuL14·3H_2_O complex in solid state. The marks in solid lines show the weight loss percentage and the temperature at each decomposition step. The dashed line presents the derivative of weight loss (%/min), Figure S6: Thermal decomposition analysis of MnL14·H_2_O complex in solid state. The marks in solid lines show the weight loss percentage and the temperature at each decomposition step. The dashed line presents the derivative of weight loss (%/min), Figure S7: Thermal decomposition analysis of FeL14·NO_3_·3H_2_O complex in solid state. The marks in solid lines show the weight loss percentage and the temperature at each decomposition step. The dashed line presents the derivative of weight loss (%/min), Figure S8: FTIR and Raman spectra of receptor H_2_L14, Figure S9: FTIR and Raman spectra of CuL14 metal complex, Figure S10: FTIR and Raman spectra of MnL14 metal complex, Figure S11: FTIR and Raman spectra of FeL14 metal complex, Figure S12: Raman spectra of the receptor H_2_L14 in presence of DPPH free radicals, Figure S13: Raman spectra of CuL14 metal complex in presence of DPPH free radicals, Figure S14: Raman spectra of MnL14 metal complex in presence of DPPH free radicals, Table S1: Crystal data and structure refinement for H_2_L14, Table S2: Fractional atomic coordinates (×10^4^) and equivalent isotropic displacement parameters (Å^2^ × 10^3^) for H_2_L14 U_eq_ is defined as 1/3 of the trace of the orthogonalized U_IJ_ tensor, Table S3: Anisotropic displacement parameters (Å^2^ × 10^3^) for H_2_L14. The anisotropic displacement factor exponent takes the form: −2π^2^[h^2^a*^2^U_11_+2hka*b*U_12_^+^], Table S4: Bond lengths for H_2_L14, Table S5. Bond angles for H_2_L14, Table S6: Hydrogen atom coordinates (Å × 10^4^) and isotropic displacement parameters (Å^2^ × 10^3^) for H_2_L14, Table S7: H bonds in molecular structure of H_2_L14.
