# Supplementary material for: Syntheses, Characterization, and Antioxidant Evaluation of Cu2+, Mn2+, and Fe3+ Complexes with a 14 Membered EDTA-Derived Macrocycle
Source: Molecules. 2019 Oct 1;24(19):3556. doi: 10.3390/molecules24193556 (PMC6804257; doi:10.3390/molecules24193556)
Supplement: Supplementary file 1 [file molecules-24-03556-s001.zip › Revised supp mat molecules-595912.docx]

*Supporting Material*

Syntheses, Characterization and Antioxidant Evaluation of Cu^2+^, Mn^2+^ and Fe^3+^ Complexes with 14- Membered EDTA-Derived Macrocycle

Yedith Soberanes ^1,3^, Rosa Elena Navarro ^1^, Motomichi Inoue ^1^, Enrique F. Velázquez-Contreras ^1^, Melissa Beltran Torres ^1^, Gustavo Lugo ^2^, Rogerio R. Sotelo-Mundo ^3^, Alex J. Salazar-Medina ^4,^*

^1^ Departamento de Investigación en Polímeros y Materiales, Universidad de Sonora, Hermosillo Sonora, 83000 México

^2^ Departamento de Ingeniería Química, Universidad de Sonora, Hermosillo, Sonora, 83000 México

^3^ Laboratorio de Estructura Biomolecular, Centro de Investigación en Alimentación y Desarrollo A.C., Hermosillo, Sonora, 83304 México

^4^ Cátedras CONACYT- Departamento de Investigación en Polímeros y Materiales, Universidad de Sonora, Hermosillo, Sonora, 83000 México

***** Correspondence: [alex.salazar@unison.mx](mailto:alex.salazar@unison.mx); Tel.: +52-662-259-2161 (A.J.S.M.)


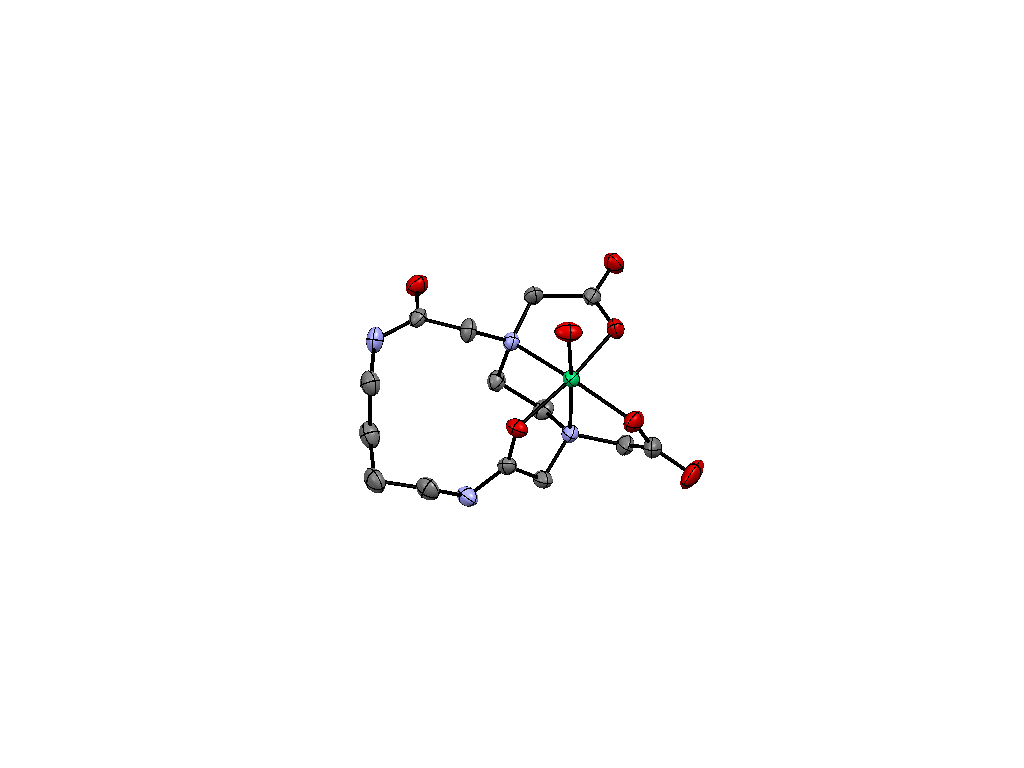


**Figure S1.** Crystal structure reported for NiL14 complex. Carbons are represented in gray, oxygens in red, nitrogens in violet and nickel in green. (Inoue, 1998) [[7](#_ENREF_6)].


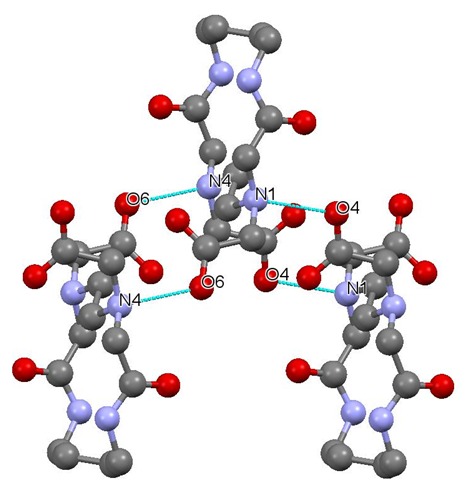


**Figure S2.** Trimer formed by hydrogen bonds in the molecular structure of H_2_L14. Atoms are drawn at the 50% probability level.

**
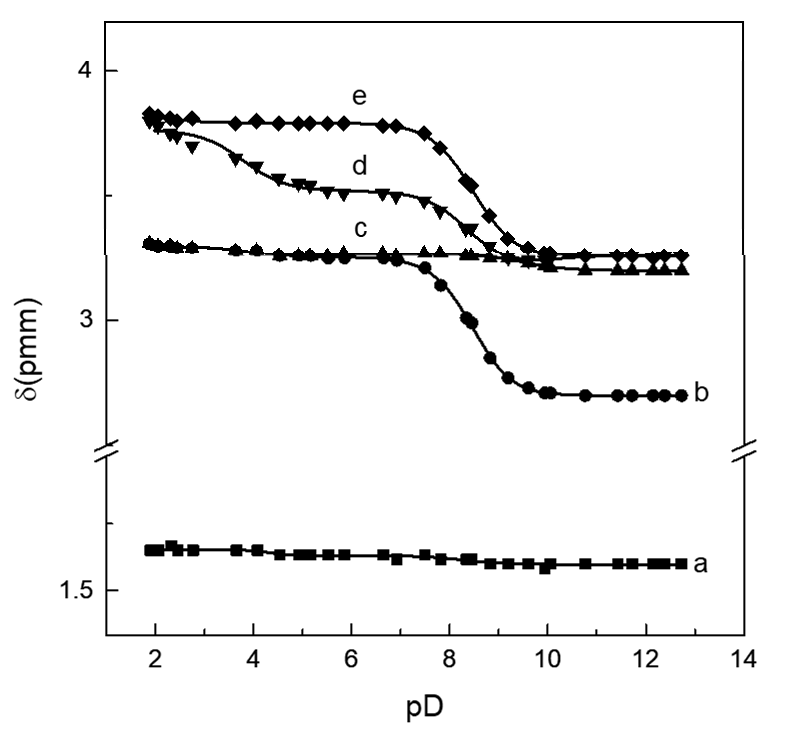
**

**
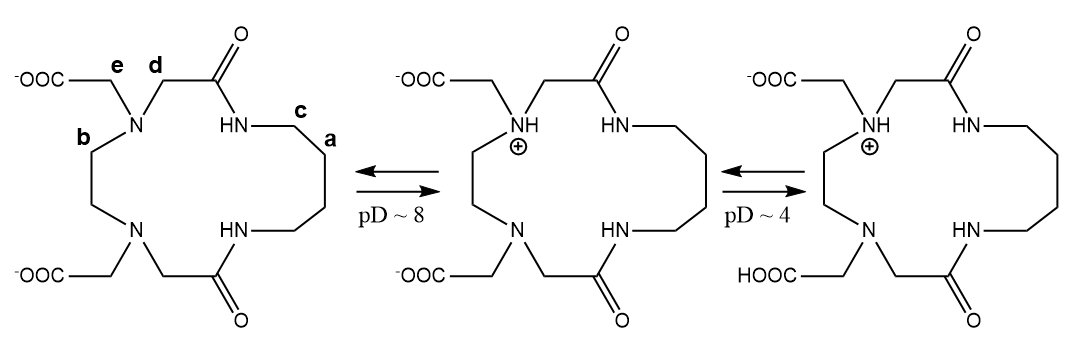
**

**Figure S3.** pD dependence of ^1^H NMR chemical shifts, δ referenced to DSS, of the L14 receptor. The solid lines are drawn as an aid to visualizing the trends of the chemical shifts. Minimal amounts of KOD and DCl were used to adjust pD.

**

**

**Figure S4.** Thermal decomposition analysis of L14 receptor in solid state. The marks in solid lines show the weight loss percentage and the temperature at each decomposition step. The dashed line presents the derivative of weight loss (%/min).

**

**

**Figure S5.** Thermal decomposition analysis of CuL14•3H_2_O complex in solid state. The marks in solid lines show the weight loss percentage and the temperature at each decomposition step. The dashed line presents the derivative of weight loss (%/min).





**Figure S6.** Thermal decomposition analysis of MnL14•H_2_O complex in solid state. The marks in solid lines show the weight loss percentage and the temperature at each decomposition step. The dashed line presents the derivative of weight loss (%/min).





**Figure S7.** Thermal decomposition analysis of FeL14•NO_3_•3H_2_O complex in solid state. The marks in solid lines show the weight loss percentage and the temperature at each decomposition step. The dashed line presents the derivative of weight loss (%/min).

**
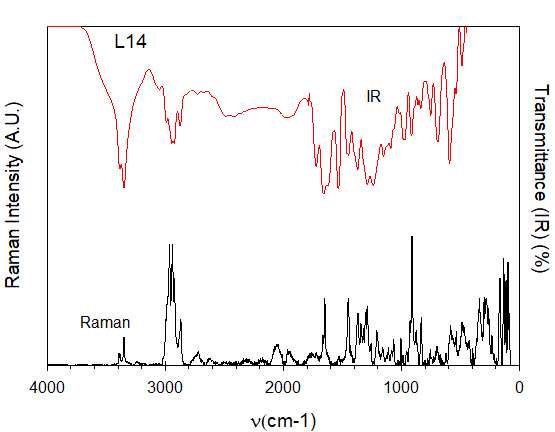
**

**Figure S8.** FTIR and Raman spectra of receptor H_2_L14.


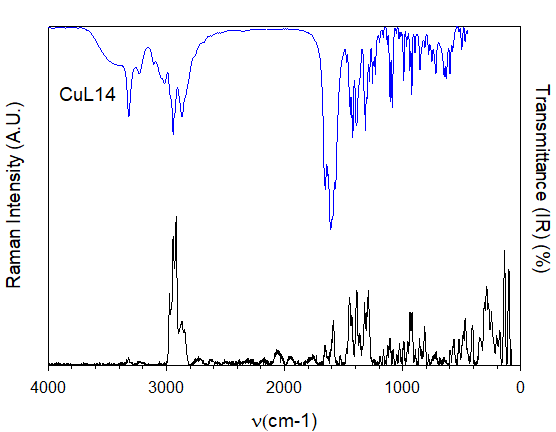


**Figure S9.** FTIR and Raman spectra of CuL14 metal complex.


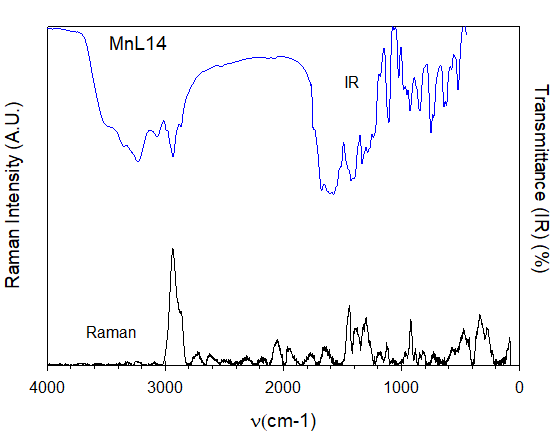


**Figure S10.** FTIR and Raman spectra of MnL14 metal complex.


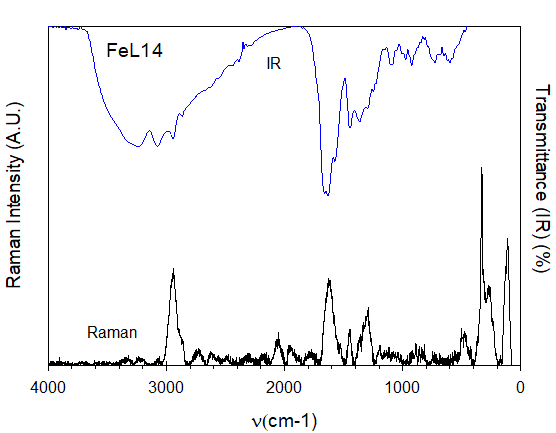


**Figure S11.** FTIR and Raman spectra of FeL14 metal complex.





**Figure S12.** Raman spectra of the receptor L14 in presence of DPPH free radical.





**Figure S13.** Raman spectra of CuL14 metal complex in presence of DPPH free radical.





**Figure S14.** Raman spectra of MnL14 metal complex in presence of DPPH free radical.

| **Table S1.**Crystal data and structure refinement for H_2_L14. | |
| --- | --- |
| Formula | C_14_H_23.5_N_4_O_6_ |
| MW (g mol^-1^) | 343.87 |
| *T* (K) | 119.95 |
| Space group | *P-1* |
| Radiation | Cu*K*_α_ |
| *a* (Å) | 9.5046(4) |
| *b* (Å) | 9.7081(4) |
| *c* (Å) | 10.1465(4) |
| *α* (deg) | 92.038(2) |
| *β* (deg) | 116.028(2) |
| *γ* (deg) | 109.223(2) |
| *V* (Å^3^) | 776.37(6) |
| *Z* | 2 |
| *µ* (mm^-1^) | 0.976 |
| *ρ*_calcd_ (g cm^-3^) | 1.471 |
| *R_1_* (*F*_o_ > 4*σF*_o_) | 0.0516 |
| *wR_2_* (all data) | 0.1524 |
| *GOF* | 1.069 |
| REFCODE/CCDC |  |

| **Table S2.** Fractional Atomic Coordinates (×10^4^) and Equivalent Isotropic Displacement Parameters (Å^2^×10^3^) for H_2_L14 U_eq_ is defined as 1/3 of of the trace of the orthogonalised U_IJ_ tensor. | | | | |
| --- | --- | --- | --- | --- |
| **Atom** | ***x*** | ***y*** | ***z*** | **U(eq)** |
| O4 | 8069(2) | 5858.7(19) | 5394(2) | 18.5(4) |
| O1 | 2142(2) | 5338.2(18) | 7453(2) | 17.4(4) |
| O2 | 8083(2) | 9784.3(19) | 10616.2(19) | 18.1(4) |
| O6 | 1860(2) | 9044.5(19) | 2316.0(18) | 17.0(4) |
| O5 | 2707(3) | 9840(2) | 4736(2) | 23.8(4) |
| N4 | 7374(2) | 8076(2) | 7889(2) | 12.5(4) |
| N1 | 2638(2) | 7012(2) | 5346(2) | 12.7(4) |
| N3 | 7733(3) | 7633(2) | 11471(2) | 17.0(5) |
| N2 | 2285(3) | 7349(2) | 8781(2) | 16.4(5) |
| O3 | 6740(3) | 5060(2) | 6734(3) | 42.0(6) |
| C8 | 8374(3) | 7815(3) | 9401(3) | 13.6(5) |
| C7 | 8049(3) | 8496(3) | 10557(3) | 13.8(5) |
| C10 | 4505(3) | 7771(3) | 5945(3) | 16.9(5) |
| C11 | 7944(3) | 7678(3) | 6828(3) | 14.5(5) |
| C2 | 2165(3) | 6614(3) | 7567(3) | 13.5(5) |
| C5 | 5548(4) | 8160(3) | 11847(3) | 20.5(6) |
| C13 | 1764(3) | 7240(3) | 3784(3) | 16.2(5) |
| C1 | 1998(3) | 7464(3) | 6314(3) | 16.4(5) |
| C14 | 2154(3) | 8857(3) | 3642(3) | 15.2(5) |
| C12 | 7516(3) | 6042(3) | 6307(3) | 16.8(5) |
| C4 | 4163(3) | 6622(3) | 10986(3) | 19.3(5) |
| C9 | 5509(3) | 7327(3) | 7375(3) | 15.4(5) |
| C6 | 7322(4) | 8134(3) | 12586(3) | 20.3(5) |
| C3 | 2418(4) | 6667(3) | 10072(3) | 20.1(5) |

| **Table S3.** Anisotropic Displacement Parameters (Å^2^×10^3^) for H_2_L14. The Anisotropic displacement factor exponent takes the form: -2π^2^[h^2^a*^2^U_11_+2hka*b*U_12_+]. | | | | | | |
| --- | --- | --- | --- | --- | --- | --- |
| **Atom** | **U_11_** | **U_22_** | **U_33_** | **U_23_** | **U_13_** | **U_12_** |
| O4 | 25.1(10) | 16.2(9) | 20.9(9) | 4.0(7) | 15.1(8) | 10.0(7) |
| O1 | 22.1(9) | 12.2(9) | 20.2(9) | 4.6(7) | 12.0(8) | 6.4(7) |
| O2 | 23.6(10) | 13.3(9) | 18.3(9) | 3.1(7) | 10.1(8) | 7.9(7) |
| O6 | 23.2(9) | 14.4(8) | 12.9(8) | 4.9(6) | 7.2(7) | 8.3(7) |
| O5 | 37.7(11) | 15.9(9) | 15.7(9) | 4.0(7) | 11.9(8) | 9.1(8) |
| N4 | 14.2(10) | 12.0(10) | 12.4(10) | 4.4(7) | 6.4(8) | 6.1(8) |
| N1 | 14.5(10) | 12.6(10) | 12.2(10) | 4.8(7) | 6.0(8) | 6.8(8) |
| N3 | 22.1(11) | 14.1(11) | 16.9(10) | 4.9(8) | 9.4(9) | 9.1(8) |
| N2 | 21.5(11) | 12.1(11) | 20.0(11) | 3.8(8) | 12.1(9) | 8.5(8) |
| O3 | 78.6(18) | 18.1(11) | 57.2(15) | 11.8(10) | 58.2(14) | 14.3(11) |
| C8 | 16.6(12) | 12.2(12) | 13.2(11) | 5.6(9) | 6.4(10) | 7.8(9) |
| C7 | 10.8(11) | 15.3(12) | 10.0(11) | 0.8(9) | 1.0(9) | 4.6(9) |
| C10 | 12.8(12) | 18.4(13) | 18.2(12) | 7.3(10) | 6.1(10) | 5.8(10) |
| C11 | 16.8(13) | 16.2(12) | 13.6(12) | 4.7(9) | 8.6(10) | 7.9(10) |
| C2 | 10.3(11) | 12.0(11) | 17.4(12) | 4.0(9) | 6.9(9) | 2.9(9) |
| C5 | 28.4(14) | 20.3(13) | 17.3(12) | 3.1(10) | 13.9(11) | 10.5(11) |
| C13 | 17.0(13) | 14.4(12) | 12.1(12) | 4.4(9) | 4.3(10) | 3.7(9) |
| C1 | 18.2(13) | 16.9(13) | 20.2(13) | 8.2(10) | 11.5(11) | 10.2(10) |
| C14 | 14.2(12) | 15.3(12) | 15.2(12) | 3.9(9) | 6.1(10) | 5.9(9) |
| C12 | 21.9(13) | 15.5(12) | 16.8(12) | 5.5(9) | 10.7(11) | 9.5(10) |
| C4 | 28.1(14) | 16.4(13) | 17.3(12) | 6.4(10) | 14.0(11) | 8.1(11) |
| C9 | 13.4(12) | 18.1(13) | 14.3(12) | 4.6(9) | 6.1(10) | 6.2(10) |
| C6 | 27.8(14) | 21.8(13) | 13.0(12) | 4.9(10) | 11.1(11) | 9.8(11) |
| C3 | 28.2(14) | 18.5(13) | 19.5(13) | 4.6(10) | 17.3(12) | 7.5(11) |

| **Table S4.** Bond Lengths for H_2_L14. | | | | | | |
| --- | --- | --- | --- | --- | --- | --- |
| **Atom** | **Atom** | **Length/Å** |  | **Atom** | **Atom** | **Length/Å** |
| O4 | C12 | 1.282(3) |  | N3 | C6 | 1.464(3) |
| O1 | C2 | 1.232(3) |  | N2 | C2 | 1.341(3) |
| O2 | C7 | 1.239(3) |  | N2 | C3 | 1.464(3) |
| O6 | C14 | 1.282(3) |  | O3 | C12 | 1.220(3) |
| O5 | C14 | 1.226(3) |  | C8 | C7 | 1.519(3) |
| N4 | C8 | 1.493(3) |  | C10 | C9 | 1.517(3) |
| N4 | C11 | 1.491(3) |  | C11 | C12 | 1.514(3) |
| N4 | C9 | 1.502(3) |  | C2 | C1 | 1.520(3) |
| N1 | C10 | 1.491(3) |  | C5 | C4 | 1.526(4) |
| N1 | C13 | 1.497(3) |  | C5 | C6 | 1.525(4) |
| N1 | C1 | 1.482(3) |  | C13 | C14 | 1.517(3) |
| N3 | C7 | 1.336(3) |  | C4 | C3 | 1.521(4) |

| **Table S5.** Bond Angles for H_2_L14. | | | | | | | | |
| --- | --- | --- | --- | --- | --- | --- | --- | --- |
| **Atom** | **Atom** | **Atom** | **Angle/˚** |  | **Atom** | **Atom** | **Atom** | **Angle/˚** |
| C8 | N4 | C9 | 110.23(18) |  | O1 | C2 | C1 | 122.4(2) |
| C11 | N4 | C8 | 110.45(18) |  | N2 | C2 | C1 | 114.5(2) |
| C11 | N4 | C9 | 115.46(19) |  | C6 | C5 | C4 | 112.8(2) |
| C10 | N1 | C13 | 108.57(19) |  | N1 | C13 | C14 | 115.2(2) |
| C1 | N1 | C10 | 114.20(19) |  | N1 | C1 | C2 | 113.1(2) |
| C1 | N1 | C13 | 112.14(19) |  | O6 | C14 | C13 | 113.5(2) |
| C7 | N3 | C6 | 121.6(2) |  | O5 | C14 | O6 | 126.1(2) |
| C2 | N2 | C3 | 121.0(2) |  | O5 | C14 | C13 | 120.4(2) |
| N4 | C8 | C7 | 110.63(19) |  | O4 | C12 | C11 | 111.5(2) |
| O2 | C7 | N3 | 123.5(2) |  | O3 | C12 | O4 | 126.3(2) |
| O2 | C7 | C8 | 120.2(2) |  | O3 | C12 | C11 | 122.2(2) |
| N3 | C7 | C8 | 116.3(2) |  | C3 | C4 | C5 | 113.2(2) |
| N1 | C10 | C9 | 112.5(2) |  | N4 | C9 | C10 | 110.06(19) |
| N4 | C11 | C12 | 118.0(2) |  | N3 | C6 | C5 | 111.4(2) |
| O1 | C2 | N2 | 123.1(2) |  | N2 | C3 | C4 | 112.0(2) |

| **Table S6.** Hydrogen Atom Coordinates (Å×10^4^) and Isotropic Displacement Parameters (Å^2^×10^3^) for H_2_L14. | | | | |
| --- | --- | --- | --- | --- |
| **Atom** | ***x*** | ***y*** | ***z*** | **U(eq)** |
| H1A | 850(40) | 7270(30) | 5700(30) | 9(6) |
| H13A | 2040(40) | 6680(30) | 3160(30) | 13(7) |
| H13B | 600(50) | 6730(40) | 3420(40) | 30(9) |
| H6A | 7400(40) | 7440(30) | 13260(40) | 20(7) |
| H3 | 7650(40) | 6700(40) | 11310(30) | 16(7) |
| H11A | 9160(40) | 8230(40) | 7310(40) | 22(8) |
| H2 | 2320(40) | 8210(40) | 8800(40) | 31(9) |
| H11B | 7500(40) | 8100(30) | 5940(30) | 18(7) |
| H8A | 8130(30) | 6790(30) | 9380(30) | 6(6) |
| H3A | 2160(40) | 7210(30) | 10680(40) | 21(8) |
| H3B | 1510(40) | 5720(30) | 9720(30) | 16(7) |
| H5A | 5490(40) | 8870(40) | 11180(40) | 22(8) |
| H10A | 4780(40) | 8790(40) | 6060(30) | 19(7) |
| H8B | 9580(40) | 8300(40) | 9660(40) | 27(8) |
| H9A | 5350(40) | 7740(30) | 8190(30) | 20(7) |
| H4A | 4090(40) | 5960(40) | 11610(40) | 23(8) |
| H1B | 2540(40) | 8490(40) | 6680(40) | 24(8) |
| H6B | 8240(40) | 9120(40) | 13230(40) | 22(8) |
| H9B | 5200(40) | 6250(40) | 7220(30) | 20(8) |
| H4B | 4340(40) | 6090(40) | 10240(40) | 28(8) |
| H5B | 5360(40) | 8490(40) | 12610(40) | 30(9) |
| H10B | 4720(40) | 7480(30) | 5150(40) | 22(8) |
| H4 | 7660(50) | 9250(50) | 7950(50) | 61(13) |
| H1 | 2370(70) | 5760(60) | 5200(60) | 7(12) |

.

| **Table S7.** H bonds in molecular structure of H_2_L14. | | | | |
| --- | --- | --- | --- | --- |
| **X-H···Y** | **X-H** | **H···Y** | **X···Y** | **X-H···Y** |
| N4-H4···O6' | 1.074(5) | 1.632(5) | 2.687(3) | 165.43(5) |
| N1'-H1'···O4 | 1.146(6) | 1.512(6) | 2.637(3) | 166.18(5) |
